# Supplementary material for: Genome-wide identification and analysis of Lateral Organ Boundaries Domain (LBD) transcription factor gene family in melon (Cucumis melo L.)
Source: PeerJ. 2023 Sep 29;11:e16020. doi: 10.7717/peerj.16020 (PMC10544307; doi:10.7717/peerj.16020)
Supplement: Supplemental Information 1 [file peerj-11-16020-s001.docx]

**Table S1. Primers sequences used for qRT-PCR.**

| **Gene Name** | **Left Primer** | **Right Primer** | **Product Size (bp)** |
| --- | --- | --- | --- |
| ***CmLBD01*** | ATGATGAGTGGGAGCTGCAA | GAACTTACTGCATCGCTCCG | 221 |
| ***CmLBD03*** | ATCACTCACCACCACCACAA | CGTGAGGGAAATAAGGAGCG | 160 |
| ***CmLBD14*** | CTGCAAATTTCTCCGGCGAA | GACAGCGTCTTCTCTCTGGT | 163 |
| ***CmLBD16*** | TCAACGCTGGTCCTGATCAT | AGCACCTTTAAGTACGGCCT | 161 |
| ***CmLBD18*** | TGTCCTTTATCTCCGCCGTT | GTTGAGAATGGTATCCGCCG | 164 |
| ***Actin*** | TGAGCAAGGAGATTACAGCATTGG | CATACTCTGCCTTCGCAATCCAC | 150 |
