# Supplementary material for: Genome-wide identification and analysis of Lateral Organ Boundaries Domain (LBD) transcription factor gene family in melon (Cucumis melo L.)
Source: PeerJ. 2023 Sep 29;11:e16020. doi: 10.7717/peerj.16020 (PMC10544307; doi:10.7717/peerj.16020)
Supplement: Supplemental Information 2 [file peerj-11-16020-s002.docx]

**Table S2. The detailed information of CmLBD genes in Cucumis melo.**

| **Gene Name** | **Gene ID** | **Chromosome distribution** | **Location** | **Size (aa)** | **ORF (bp)** | **MW (kDA)** | **pI** | **GRAVY** | **Predicted Location** |
| --- | --- | --- | --- | --- | --- | --- | --- | --- | --- |
| ***CmLBD01*** | MELO3C012624 | chr01 | 21531991-21532272 | 160 | 483 | 17.23 | 8.14 | -0,159 | Nucleus |
| ***CmLBD02*** | MELO3C012741 | chr01 | 24021951-24022241 | 184 | 555 | 20.24 | 6.28 | 0,014 | Nucleus |
| ***CmLBD03*** | MELO3C024387 | chr01 | 36511709-36512445 | 352 | 1059 | 39.56 | 6.01 | -0,619 | Nucleus |
| ***CmLBD04*** | MELO3C017305 | chr02 | 24492109-24492298 | 245 | 738 | 26.76 | 8.72 | -0,24 | Nucleus |
| ***CmLBD05*** | MELO3C026269 | chr02 | 26360912-26361469 | 185 | 558 | 20.36 | 7.63 | -0,405 | Nucleus |
| ***CmLBD06*** | MELO3C008309 | chr03 | 3434503-3435021 | 172 | 519 | 19.11 | 8.10 | -0,322 | Nucleus |
| ***CmLBD07*** | MELO3C010752 | chr03 | 31106153-31106734 | 193 | 582 | 21.54 | 7.99 | -0,186 | Nucleus |
| ***CmLBD08*** | MELO3C011135 | chr03 | 28428479-28428769 | 96 | 291 | 10.51 | 4.75 | -0,089 | Nucleus |
| ***CmLBD09*** | MELO3C019995 | chr03 | 21023714-21023727 | 277 | 834 | 30.18 | 8.75 | -0,226 | Nucleus |
| ***CmLBD10*** | MELO3C009989 | chr04 | 27447473-27448006 | 177 | 534 | 19.52 | 7.58 | -0,64 | Nucleus |
| ***CmLBD11*** | MELO3C012908 | chr04 | 14403798-14404076 | 243 | 732 | 27.01 | 6.04 | -0,5 | Nucleus |
| ***CmLBD12*** | MELO3C024038 | chr04 | 23347131-23347463 | 236 | 711 | 25.57 | 7.06 | -0,25 | Nucleus |
| ***CmLBD13*** | MELO3C030713 | chr04 | 29177450-29177776 | 224 | 675 | 23.98 | 6.49 | -0,215 | Nucleus |
| ***CmLBD14*** | MELO3C008779 | chr05 | 19220919-19221172 | 160 | 483 | 17.88 | 9.10 | -0,48 | Nucleus |
| ***CmLBD15*** | MELO3C006183 | chr06 | 1560563-1560928 | 231 | 696 | 24.95 | 8.93 | -0,216 | Nucleus |
| ***CmLBD16*** | MELO3C023802 | chr06 | 19389817-19390535 | 362 | 1089 | 39.74 | 9.11 | -0,15 | Nucleus |
| ***CmLBD17*** | MELO3C031638 | chr06 | 6882511-6882663 | 152 | 459 | 16.93 | 8.16 | -0,102 | Nucleus |
| ***CmLBD18*** | MELO3C016808 | chr07 | 1953906-1954089 | 203 | 612 | 22.23 | 6.50 | -0,202 | Nucleus |
| ***CmLBD19*** | MELO3C007098 | chr08 | 780445-781167 | 240 | 723 | 27.29 | 7.62 | -0,634 | Nucleus |
| ***CmLBD20*** | MELO3C007620 | chr08 | 4167441-4167955 | 232 | 699 | 25.56 | 7.58 | -0,39 | Nucleus |
| ***CmLBD21*** | MELO3C021578 | chr09 | 4033375-4033872 | 165 | 498 | 18.20 | 7.66 | -0,524 | Nucleus |
| ***CmLBD22*** | MELO3C021964 | chr09 | 2489335-2490129 | 265 | 795 | 29.99 | 5.00 | -0,57 | Nucleus |
| ***CmLBD23*** | MELO3C025504 | chr09 | 6398051-6398173 | 100 | 303 | 11.43 | 9.43 | -0,102 | Nucleus |
| ***CmLBD24*** | MELO3C011730 | chr10 | 5379749-5380066 | 209 | 630 | 23.14 | 8.39 | -0,216 | Nucleus |
| ***CmLBD25*** | MELO3C012250 | chr10 | 1706207-1706396 | 191 | 573 | 20.81 | 8.90 | -0,375 | Nucleus |
| ***CmLBD26*** | MELO3C018380 | chr10 | 18000454-18000768 | 167 | 504 | 18.25 | 6.48 | -0,053 | Nucleus |
| ***CmLBD27*** | MELO3C020121 | chr10 | 13055338-13055351 | 244 | 735 | 26.81 | 8.61 | -0,491 | Nucleus |
| ***CmLBD28*** | MELO3C023880 | chr10 | 7203948-7204241 | 221 | 666 | 24.27 | 4.45 | -0,051 | Nucleus |
| ***CmLBD29*** | MELO3C019314 | chr11 | 11426653-11427369 | 238 | 714 | 26.82 | 8.88 | -0,772 | Nucleus |
| ***CmLBD30*** | MELO3C020944 | chr11 | 2643291-2644223 | 310 | 933 | 34.94 | 7.12 | -0,792 | Nucleus |
| ***CmLBD31*** | MELO3C022505 | chr11 | 34151365-34151706 | 269 | 810 | 29.54 | 9.04 | -0,373 | Nucleus |
| ***CmLBD32*** | MELO3C025701 | chr11 | 27430942-27431100 | 211 | 636 | 23.86 | 5.16 | 0,012 | Nucleus |
| ***CmLBD33*** | MELO3C025742 | chr11 | 28003845-28004093 | 261 | 786 | 28.82 | 8.72 | -0,125 | Nucleus |
| ***CmLBD34*** | MELO3C035136 | chr11 | 24854465-24854647 | 72 | 219 | 8.13 | 9.10 | -0,188 | Nucleus |
| ***CmLBD35*** | MELO3C002290 | chr12 | 25094369-25094872 | 167 | 504 | 18.81 | 8.52 | -0,359 | Nucleus |
| ***CmLBD36*** | MELO3C005009 | chr12 | 4697982-4698335 | 239 | 720 | 26.41 | 7.02 | -0,003 | Nucleus |
| ***CmLBD37*** | MELO3C005013 | chr12 | 4640408-4640740 | 177 | 534 | 20.03 | 7.60 | -0,265 | Nucleus |
| ***CmLBD38*** | MELO3C021789 | chr12 | 17453413-17453595 | 170 | 513 | 18.49 | 8.32 | -0,281 | Nucleus |
| ***CmLBD39*** | MELO3C000068 | / | 8845194-8845538 | 131 | 396 | 13.69 | 9.33 | 0,127 | Nucleus |
| ***CmLBD40*** | MELO3C000076 | / | 8834266-8834565 | 99 | 300 | 10.95 | 5.67 | -0,268 | Nucleus |
