# Supplementary material for: Genome-wide identification and analysis of Lateral Organ Boundaries Domain (LBD) transcription factor gene family in melon (Cucumis melo L.)
Source: PeerJ. 2023 Sep 29;11:e16020. doi: 10.7717/peerj.16020 (PMC10544307; doi:10.7717/peerj.16020)
Supplement: Supplemental Information 3 — C stage (climacteric stage), G stage (growing stage), R stage (ripening stage), P stage (post-climacteric stage). Fold changes are given in log2-based numbers (↑ up-regulation, ↓ down-regulation). [file peerj-11-16020-s003.docx]

**Table S3. Differentially expressed CmLBD genes at different developmental stages in melon.** C stage (climacteric stage), G stage (growing stage), R stage (ripening stage), P stage (post-climacteric stage). Fold changes are given in log2-based numbers (↑ up-regulation, ↓ down-regulation).

| Comparison | Gene name | Fold change |  | *P* value |
| --- | --- | --- | --- | --- |
| C1  (climacteric - growing stage) | *CmLBD06* | 2,71 | **↑** | 6,52E-06 |
|  | *CmLBD07* | -3,46 | **↓** | 2,98E-11 |
|  | *CmLBD09* | 9,86 | **↑** | 3,27E-14 |
|  | *CmLBD18* | 6,60 | **↑** | 3,18E-69 |
|  | *CmLBD21* | 7,52 | **↑** | 8,89E-06 |
|  | *CmLBD24* | 5,14 | **↑** | 1,18E-02 |
|  | *CmLBD27* | 5,70 | **↑** | 6,09E-04 |
|  | *CmLBD34* | 8,60 | **↑** | 2,77E-11 |
|  | *CmLBD37* | -2,67 | **↓** | 5,89E-05 |
|  | *CmLBD38* | 6,14 | **↑** | 4,55E-06 |
|  | *CmLBD40* | 8,78 | **↑** | 2,20E-12 |
| C3  (climacteric - ripening stage) | *CmLBD04* | -1,67 | **↓** | 2,47E-05 |
|  | *CmLBD18* | 5,05 | **↑** | 2,26E-37 |
|  | *CmLBD37* | -1,69 | **↓** | 1,21E-02 |
| C4  (growing - post-climacteric stage) | *CmLBD03* | -1,79 | **↓** | 1,03E-02 |
|  | *CmLBD06* | -4,03 | **↓** | 4,28E-13 |
|  | *CmLBD07* | 3,48 | **↑** | 1,03E-44 |
|  | *CmLBD09* | -9,84 | **↓** | 9,70E-15 |
|  | *CmLBD18* | -5,36 | **↓** | 3,11E-39 |
|  | *CmLBD21* | -7,51 | **↓** | 2,86E-06 |
|  | *CmLBD24* | -5,12 | **↓** | 1,21E-02 |
|  | *CmLBD27* | -5,69 | **↓** | 8,73E-04 |
|  | *CmLBD34* | -8,59 | **↓** | 2,27E-11 |
|  | *CmLBD37* | 2,80 | **↑** | 1,61E-07 |
|  | *CmLBD38* | -9,31 | **↓** | 1,66E-10 |
|  | *CmLBD40* | -8,77 | **↓** | 2,57E-12 |
| C5  (growing - ripening stage) | *CmLBD06* | -2,64 | **↓** | 3,61E-06 |
|  | *CmLBD07* | 4,56 | **↑** | 2,70E-25 |
|  | *CmLBD09* | -6,62 | **↓** | 3,58E-12 |
|  | *CmLBD18* | -1,52 | **↓** | 5,67E-09 |
|  | *CmLBD21* | -4,84 | **↓** | 1,23E-03 |
|  | *CmLBD27* | -5,99 | **↓** | 7,01E-04 |
|  | *CmLBD34* | -8,89 | **↓** | 8,71E-12 |
|  | *CmLBD40* | -7,20 | **↓** | 1,03E-10 |
| C6  (post-climacteric - ripening stage) | *CmLBD03* | 1,97 | **↑** | 6,32E-03 |
|  | *CmLBD04* | -2,21 | **↓** | 6,30E-07 |
|  | *CmLBD18* | 3,81 | **↑** | 2,83E-15 |
|  | *CmLBD37* | -1,83 | **↓** | 5,15E-04 |
|  | *CmLBD38* | 6,44 | **↑** | 1,67E-02 |
